# Prescribing “placebo treatments”: results of national survey of US internists and rheumatologists

**DOI:** 10.1136/bmj.a1938

**Published:** 2008-10-23

**Authors:** Jon C Tilburt, Ezekiel J Emanuel, Ted J Kaptchuk, Farr A Curlin, Franklin G Miller

**Affiliations:** 1 Department of Bioethics, National Institutes of Health, Bethesda, MD 20892, USA; 2 Osher Institute, Harvard Medical School, 401 Park Dr, Boston, MA 02215; 3 Section of Internal Medicine and the McClean Center for Clinical Medical Ethics, University of Chicago, 5841 S Maryland Ave, MC 2007 Chicago, IL 60637

## Abstract

**Objective** To describe the attitudes and behaviours regarding placebo treatments, defined as a treatment whose benefits derive from positive patient expectations and not from the physiological mechanism of the treatment itself.

**Design** Cross sectional mailed survey.

**Setting** Physicians’ clinical practices.

**Participants** 1200 practising internists and rheumatologists in the United States.

**Main outcome measures** Investigators measured physicians’ self reported behaviours and attitudes concerning the use of placebo treatments, including measures of whether they would use or had recommended a “placebo treatment,” their ethical judgments about the practice, what they recommended as placebo treatments, and how they typically communicate with patients about the practice.

**Results** 679 physicians (57%) responded to the survey. About half of the surveyed internists and rheumatologists reported prescribing placebo treatments on a regular basis (46-58%, depending on how the question was phrased). Most physicians (399, 62%) believed the practice to be ethically permissible. Few reported using saline (18, 3%) or sugar pills (12, 2%) as placebo treatments, while large proportions reported using over the counter analgesics (267, 41%) and vitamins (243, 38%) as placebo treatments within the past year. A small but notable proportion of physicians reported using antibiotics (86, 13%) and sedatives (86, 13%) as placebo treatments during the same period. Furthermore, physicians who use placebo treatments most commonly describe them to patients as a potentially beneficial medicine or treatment not typically used for their condition (241, 68%); only rarely do they explicitly describe them as placebos (18, 5%).

**Conclusions** Prescribing placebo treatments seems to be common and is viewed as ethically permissible among the surveyed US internists and rheumatologists. Vitamins and over the counter analgesics are the most commonly used treatments. Physicians might not be fully transparent with their patients about the use of placebos and might have mixed motivations for recommending such treatments.

## Introduction

The use of placebo treatments in clinical medicine is ethically controversial.^1^ ^2^ The placebo effect refers to positive clinical outcomes caused by a treatment that is not attributable to its known physical properties or mechanism of action. The placebo effect is often explained as the result of positive expectation, belief, or hope in patients derived from the clinical encounter.^3^ ^4^

Before 1960, administration of inert substances to promote placebo effects or to satisfy patients’ expectations of receiving a prescribed treatment was commonplace in medical practice.^5^ ^6^ ^7^ ^8^ ^9^ ^10^ ^11^ ^12^ ^13^ With the development of effective pharmaceutical interventions and the increased emphasis on informed consent, the use of placebo treatments in clinical care has been widely criticised. Prescribing a placebo, it is claimed, involves deception and therefore violates patients’ autonomy and informed consent.^14^ ^15^ Advocates of placebo treatments argue that promoting the placebo effect might be one of the most effective treatments available for many chronic conditions and can be accomplished without deception.^16^ ^17^ ^18^ ^19^

“Placebo treatment” is an unclear and complicated concept that lacks a standard definition. Placebo treatments include “inert” agents that have little or no pharmacological activity, such as sugar pills and saline injections given to promote positive expectation, and physiologically active agents, such as vitamins or antibiotics, that the physician prescribes solely or primarily to promote positive psychological effects.^16^ Furthermore, clinicians might recommend an “active” treatment to elicit a possible pharmacological effect and a placebo response simultaneously. Given these and other terminological difficulties,^3^ for the purposes of our research we defined a “placebo treatment” as a treatment whose benefits (in the opinion of the clinician) derive from positive patient expectations and not from the physiological mechanism of the treatment itself.

Despite the persistent controversy surrounding the use of placebo treatments, there are few systematic data concerning physicians’ attitudes towards and use of placebo treatments in the United States.^20^ ^21^ ^22^ The few contemporary surveys from other countries suggest that more than half of physicians prescribe placebo treatment.^23^ ^24^ ^25^ ^26^ ^27^

To further inform ethical discussions about the appropriateness of recommending placebo treatments, we examined the attitudes and behaviours regarding placebo treatments among a national sample of clinically active internists and rheumatologists in the US.

## Methods

### Study population

Using the 2006 American Medical Association masterfile, we randomly selected 1200 physicians listed with the primary specialties of internal medicine (600) or rheumatology (600): a group of physicians who commonly treat patients with debilitating chronic clinical conditions that are notoriously difficult to manage. Participants were screened to determine if they were currently practising. In June 2007, an independent survey research firm posted a confidential, self administered survey, a $20 (£11, €15) incentive, and a letter outlining the voluntary nature of participation. Participants were assured that their identities would not be disclosed to investigators. Those who did not respond to the first survey were sent a second six weeks later. Response rates were calculated with standard conservative survey research definitions (RR1 from the American Association for Public Opinion Research).^28^ Of the 1200 physicians who were sent questionnaires, 679 responded (overall response rate 57%), of whom 334 specialised in internal medicine (56% response rate) and 345 in rheumatology (58% response rate).

### Survey instrument

The questions on use of placebo treatment were incorporated into a survey that covered other topics related to complementary and alternative medicine (details available on request). The survey was developed through a formal process and included a review of existing surveys on the use of placebo treatments.^23^ ^24^ Because the term “placebo” and behaviours surrounding its use can be contentious, we devised a series of non-judgmental questions beginning with broad questions that avoided the term “placebo” and then gradually gained more specificity, culminating in items whose responses used a clear definition of a “placebo treatment.” By constructing a series of items in this manner we allowed respondents to describe their attitudes and experiences as accurately as possible.

The first set of three items began with a hypothetical scenario in which a dextrose tablet was shown in clinical trials to be superior to a no treatment control group (thus establishing its efficacy as a placebo treatment). To avoid biasing responses these three questions did not use the term “placebo,” “placebo treatment,” or “placebo effect.” Respondents were then asked to rate the likelihood of their personally recommending this treatment to non-diabetic patients with fibromyalgia; how often they recommend a therapy “primarily because you believe it will enhance the patient’s expectation of getting better”; and whether recommending treatments in this manner was “obligatory,” “permissible,” “permissible in rare circumstances,” or “never permissible.” Respondents were then asked to indicate which of several treatments they had used within the past year primarily as a placebo treatment, defined as a treatment whose benefits derive from positive patient expectations and not from the physiological mechanism of the treatment itself; and how they typically described placebo treatments to patients. By asking these five questions both without the term “placebo” and then using the term, we aimed to assess physicians’ practices as accurately as possible.

### Data management and analysis

All data were double entered and 100% verified. We used descriptive statistics to examine physicians’ characteristics as well as frequencies of reported behaviours and attitudes. Percentages reported are based on actual numbers of respondents. All 679 respondents answered most questions. We used multivariate logistic regression to determine if any characteristics of participants were independently associated with regularly prescribing placebo treatments. For this analysis our dependent variable was recommending treatments “primarily to promote patient expectations” at least two to three times a month based on self reporting. All analyses were conducted with STATA (version 8.0).

## Results

The mean age of the 679 respondents was 51 years (range 28-88), 73% (477/652) were men, and 81% (526/648) were white. Overall, respondents most commonly reported a group practice setting (49%, 334/679), followed by solo practice (27%, 186/679), academic (14%, 96/679) and institutional (4%, 28/679). The largest proportions of respondents practised in the south (37%, 253/679) and the north east (32%, 219/679) (table 1). Respondents and non-respondents did not differ significantly according to age, sex, race, practice setting, or specialty.

**Table 1 tbl1:** Characteristics of 679 US physicians questioned about use of placebos. Figures are numbers (percentages) of responses apart from age

Characteristics	No (%)*
Mean (range) age (years)	51 (28-88)
Male	477/652 (73)
Race/ethnicity:	
Asian	89/648 (14)
African American	14/648 (2)
White	526/648 (81)
Other	19/648 (3)
Specialty:	
Internal medicine	334/679 (49)
Rheumatology	345/679 (51)
Practice setting:	
Solo	186/679 (27)
Group	334/679 (49)
Institutional	28/679 (4)
Academic	96/679 (14)
Other	24/679 (4)
Region:	
South	253/679 (37)
North east	219/679 (32)
Midwest	108/679 (16)
West	99/679 (15)

*Percentages based on actual numbers of respondents as shown.

When asked if they would recommend a dextrose tablet for a patient with fibromyalgia if trials had shown such treatment to be superior to no treatment, most respondents (58%, 381/654) said they would be very likely or moderately likely to recommend it. Similarly, 46% (298/646) reported actually recommending a treatment primarily to promote patient expectations at least two to three times a month. The physicians’ ethical judgments were also favourable toward the use of placebo treatments, and 62% (399/642) said recommending treatments in this manner was ethically obligatory or permissible (table 2).

**Table 2 tbl2:** Attitudes and behaviours related to prescribing placebos among 679 US general internists and rheumatologists

Question and categories of response	No (%*)
How likely are you to recommend sugar pill proved to be better than no treatment for fibromyalgia?:	
Very likely	160/654 (24)
Moderately likely	221/654 (34)
Unlikely	205/654 (31)
Definitely not	68/654 (10)
How often do you recommend treatment primarily to enhance patient expectation?:	
Never	129/646 (20)
≤1/month	219/646 (34)
2-3/month	182/646 (28)
≤1/week	116/646 (18)
Is it appropriate to recommend treatment primarily to promote patients’ expectations?:	
Obligatory	19/642 (3)
Permissible	380/642 (59)
Permissible only in rare circumstance	197/642 (31)
Never permissible	46/642 (7)

*Based on actual numbers.

Within the previous year, 55% of (370/679) physicians reported having recommended at least one placebo treatment (including “active” and “inactive”). Active placebo treatments were more commonly reported, such as over the counter analgesics (41%), vitamins (38%), antibiotics (13%), and sedatives (13%). Only 2% recommended “sugar pills” and 3% saline (table 3).

**Table 3 tbl3:** Treatments used as placebo in past year and how they are described to patients among 679 US general internists and rheumatologists

Question and response items	No (%*)
Recommended as “placebo treatment” in past year:	
At least one of any type	370/679 (55)
Over counter analgesics	267/648 (41)
Vitamins	243/648 (38)
Sedatives	86/652 (13)
Antibiotics	85/644 (13)
Saline	18/623 (3)
Sugar pills	12/642 (2)
How placebo treatments are typically described to patients:	
Not used	285/637 (45)
Medicine	62/352 (18)
Placebo	18/352 (5)
Medicine with no known effects for your condition	31/352 (9)
Medicine not typically used for your condition but might benefit you	241/352 (68)

*Based on actual numbers of respondents. All 679 respondents answered most questions. Percentages reflect 352 responses of 637 respondents who deemed the question relevant. The 285 respondents who marked “irrelevant—I do not prescribe placebo treatments” were not included in these percentages.

When asked to describe how they typically introduce placebo treatments to their patients, 45% (285/637) reported never recommending placebo treatments, implying that 55% (352/637) agreed that they had recommended a placebo treatment as defined. Among these 352, about 68% (241) said they usually describe placebo treatments as “a medicine not typically used for your condition but might benefit you,” 18% (62) introduced placebo treatments as “a medicine,” and 9% (31) said they typically describe placebo treatments as “a medicine with no known effects for your condition.” Only 5% (18) reported typically describing the treatment as “a placebo” (table 3).[Table tbl3]

After we controlled for all other characteristics, neither age, sex, race, specialty, practice setting, nor region were independently and significantly associated with having recommended a placebo treatment (table 4).

**Table 4 tbl4:** Characteristics of US internists and rheumatologists independently associated with having recommended placebo treatment at least 2-3 times/month

Characteristics	Odds ratio* (95% CI)
Age	0.99 (0.97 to 1.01)
Male	0.76 (0.52 to 1.11)
White race	1.32 (0.85 to 2.06)
Specialty:	
Internal medicine	0.82 (0.58 to 1.16)
Rheumatology	—
Practice setting:	
Solo	—
Group	0.97 (0.66 to 1.43)
Institutional	0.72 (0.31 to 1.66)
Academic	0.73 (0.43 to 1.23)
Other	0.96 (0.40 to 2.35)
Region:	
North east	—
South	1.47 (0.99 to 2.16)
Midwest	0.72 (0.43 to 1.21)
West	1.01 (0.61 to 1.71)

*Based on responses of 637 observations included in model.

## Discussion

### Summary of major findings

About half of the 679 US internists and rheumatologists questioned reported prescribing placebo treatments to their patients on a regular basis. Many of these physicians were favourably disposed to recommending or prescribing placebo treatments. Most said they would prescribe a sugar pill for patients with a chronic painful condition if there was evidence of placebo efficacy, and nearly half said they had used treatments primarily to promote positive expectations in their patients more than once in a month. In addition, most US internists and rheumatologists believe that recommending treatments because they promote such expectations is ethically permissible. Once given a clear definition, over half said they had recommended at least one treatment within the past year as a “placebo treatment.” In some circumstances common innocuous treatments—such as vitamins or over the counter analgesics—are used; in other circumstances physicians use antibiotics and sedatives as placebo treatments. Furthermore, physicians most often describe placebo treatments to patients as a potentially beneficial medicine or treatment, rarely describing them as placebos.

Between 46% and 58% of US internists and rheumatologists engage in recommending placebo treatments as defined. This large empirical study examined attitudes and behaviours among practising physicians toward recommending placebo treatments and used a systematic, random, national sample. To accurately assess attitudes and behaviours relating to placebo treatments, we asked the physicians about recommending placebo treatments in four distinct ways: response to a hypothetical case, self reported behaviour without the term “placebo treatment,” self reported behaviour with the term “placebo treatment,” and inclusion of “I never use placebo treatments” as a response option in our item related to communication with patients. The first two of these were asked without introducing the term “placebo” to allow the most candid and unbiased responses. The third and fourth were asked after a careful definition of a “placebo treatment.” The similar rates across these four different measures indicate that our findings are unlikely to be the result of question framing, wording, or the specific definition of placebo treatment used.

### Relation to other studies

Our results are consistent with the findings of other studies. Recently, Sherman and Hickner surveyed a convenience sample of 231 academic physicians in the Chicago area and found that 45% had used placebo treatments in clinical practice.^22^ Indeed, 8% indicated using placebo treatment more than 10 times in the past year. A Danish survey reported that 86% of 545 general practitioners used a placebo treatment at least once within the past year, and 48% reported using placebo treatments more than 10 times in the past year.^23^ Smaller surveys from Israel, the UK, Sweden, and New Zealand report similar results.^24^ ^25^ ^26^ ^27^

### Unresolved questions raised by this study

Understanding the role of placebo treatments in contemporary medicine is complex. Promoting positive expectations in patients has historically been an important part of the physician’s therapeutic role, though the current major role of drug treatment is undeniable. Few of the physicians we surveyed recommend inert placebo treatments. The reasons for this are unclear. It might no longer be possible for physicians to write a prescription for a sugar or bread pill. Without the existence of pharmacies to create such pills, and a lack of actual pills being marketed for such use, physicians could not prescribe them routinely even if they wanted to do so. Or they might have understandable reservations about recommending so called “inactive” or “inert” placebo treatments, fearing these treatments are inherently deceptive and are not amenable to contemporary standards of informed consent.

Yet these data also suggest the desire to promote positive therapeutic expectations among patients is prevalent among the surveyed physicians. The responses suggest a preference for active placebo treatments. Physicians might have multiple, possibly conflicting, beliefs and motivations for recommending active placebo treatments. They might believe that a given benign and safe treatment, such as some indicated by our respondents (for example, vitamins and over the counter analgesics), might have some finite chance of a beneficial pharmacological effect, despite remaining unproved for the symptoms or condition at hand; or, at the very least, such treatments might promote positive expectations in patients or help the patient to feel like they are being taken care of. This perceived need to administer something in the absence of other proved effective treatments for chronic symptomatic conditions (that promotes a sense of treatment options and positive expectations in response to patients’ complaints) might motivate physicians to prescribe placebo treatments.^29^ We did not explore these complex beliefs and motives in this survey.

Recommending relatively innocuous treatments such as vitamins or over the counter analgesics to promote positive expectations might not raise serious concerns about detrimental effects to patients’ welfare. Prescribing antibiotics and sedatives when they are not medically indicated, however, could have potentially important adverse consequences for both patients and public health. In the absence of knowing the physicians’ indication or motivation for recommending placebo treatments, the interpretation of our findings remains speculative. These issues deserve further investigation to examine motivations, beliefs about the efficacy of placebo treatments, and the range of circumstances in which they are used among a broader sample of physicians.

There are many different descriptive terms used to refer to patients’ expectations of healing in clinical medicine and treatments that intersect with those expectations but “placebo treatment,” as defined and used in this study, is an understandable term that best captures this clinical phenomenon.

### Study limitations

This study has several limitations. The cross sectional, self reported design might not have accurately estimated the actual frequency of recommending placebo treatments. The moderate response rate (57%) also limits our ability to make exact estimates of the behaviour in the entire population of these groups of physicians; and our findings might not be generalisable to attitudes and behaviours in other medical specialties. Furthermore, because these items were included in a survey on complementary and alternative medicine, it is possible that the physicians who chose to respond were more favourably disposed to prescribe placebo treatments than most physicians. However, our findings are consistent with the results of other published studies concerning physicians’ use of placebo treatments.

### Conclusions

US internists and rheumatologists commonly recommend “placebo treatments.” Vitamins and over the counter analgesics are the most commonly prescribed. Physicians who use placebo treatments may not be fully transparent with their patients about their use. Whether, or under what circumstances, recommending or prescribing placebo treatments is appropriate remains a topic for ethical and policy debates.

**What is already known on this topic**

Recommending treatments to promote patients’ expectations (placebo treatments) seems to be common in several developed countries
The routine use of placebo treatments raises ethical questions about deception in clinical practice

**What this study adds**

Half of the US internal medicine and rheumatology physicians studied reported often recommending placebo treatments, most commonly vitamins, over the counter analgesics, and antibiotics
Most viewed this practice as ethically permissible
Physicians might not be fully transparent with their patients about their motivations but largely avoid prescribing sugar pills and saline

## Notes

Development and implementation of the survey was performed by the Center for Survey Research, University of Massachusetts, Boston, MA. Statistical support was provided by Summit Consulting, LLC, Washington, DC.

Ethical approval: This study was approved by the University of Massachusetts Boston Institutional Review Board and the Offices of Human Subjects Research at the National Institutes of Health. Returned questionnaires were considered to indicate informed consent.

Provenance and peer review: Not commissioned; externally peer reviewed.

## Notes

**Cite this as: ***BMJ* 2008;337:a1938
